# Author Correction: Rab25 augments cancer cell invasiveness through a β1 integrin/EGFR/VEGF-A/Snail signaling axis and expression of fascin

**DOI:** 10.1038/s12276-018-0148-4

**Published:** 2018-09-19

**Authors:** Bo Young Jeong, Kyung Hwa Cho, Kang Jin Jeong, Yun-Yong Park, Jin Man Kim, Sun Young Rha, Chang Gyo Park, Gordon B Mills, Jae-Ho Cheong, Hoi Young Lee

**Affiliations:** 1 Department of Pharmacology, College of Medicine, Konyang University, Daejeon, Korea; 2 Department of Systems Biology, The University of Texas MD Anderson Cancer Center, Houston, TX USA; 3 Department of Convergence Medicine, Asan Medical Center, University of Ulsan College of Medicine, Seoul, Korea; 4 Cancer Research Institute, Regional Cancer Center and Infection Signaling Network Research Center, Chungnam National University School of Medicine, Daejeon, Korea; 5 Divison of Medical Oncology, Yonsei Cancer Center, Yonsei University College of Medicine, Seoul, Korea; 6 Department of Surgery, Severance Hospital, Yonsei University College of Medicine, Seoul, Korea

**Correction to**: *Experimental & Molecular Medicine* (2018) **50**:e435; 10.1038/emm.2017.248; Published Online 26 January 2018.

After online publication of this article, the authors noticed errors in the Fig. 4c and Fig. 5c section.

**Figure 1:**
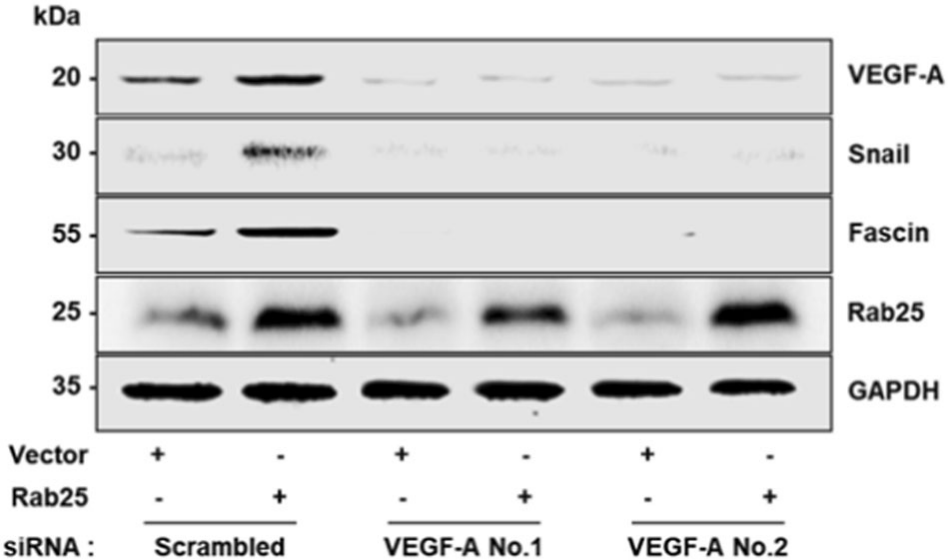
Fig. 4c

**Figure 2:**
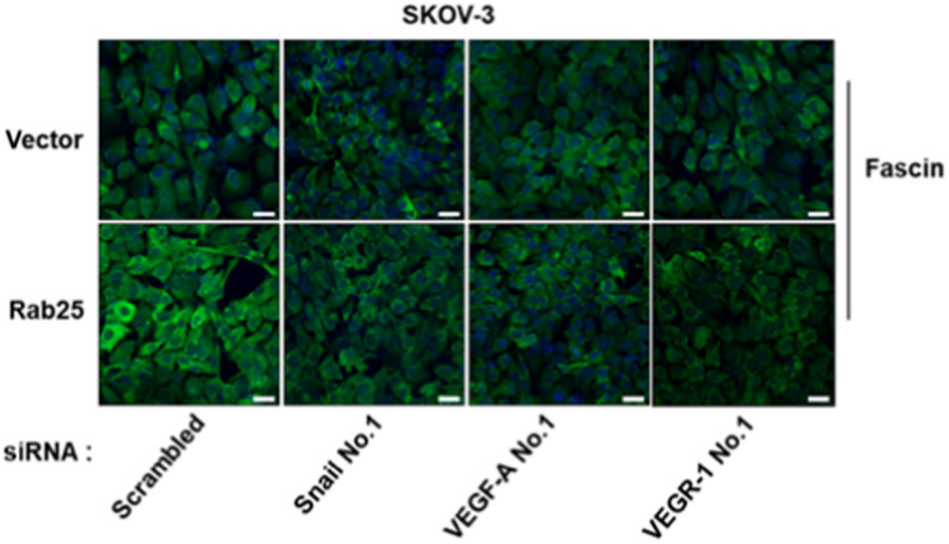
Fig. 5c

The correct statement of this article should have read as below.

In the article cited above, incorrect figures were placed in Figs. 4c and 5c.

The corrected data were printed below. These corrected results do not alter the conclusions of this article. The authors apologize for any inconvenience caused.

